# LncRNA LUADT1 sponges miR-15a-3p to upregulate Twist1 in small cell lung cancer

**DOI:** 10.1186/s12890-019-0991-7

**Published:** 2019-12-16

**Authors:** Dingxue Wang, Wenyu Wu, Wenqi Huang, Jinghui Wang, Li Luo, Dongxin Tang

**Affiliations:** 1 Department of Oncology, First Affiliated Hospital of Guizhou University of Traditional Chinese Medicine, No. 71 Baoshan North Road, Guiyang City, Guizhou Province 550001 People’s Republic of China; 2 Department of Oncology, Guihang Guiyang Hospital, Guizhou Province, Guiyang City, 550009 People’s Republic of China

**Keywords:** Small cell lung cancer, LUADT1, miR-15a-3p, Twist1

## Abstract

**Abstract:**

Lung adenocarcinoma associated transcript 1 (LUADT1) has been reported as an oncogenic long non-coding RNA (lncRNA) in lung adenocarcinoma, while its roles in small cell lung cancer (SCLC) are unknown. Our RNA interaction bioinformatics prediction showed that LUADT1 could form strong base pairing with miR-15a-3p, which is a tumor-suppressive miRNA that can target Twist1. We found that LUADT1 and Twist1 were upregulated in SCLC, while miR-15a-3p was downregulated in SCLC. However, LUADT1 was posively correlated with Twist1 but was not significnatly correlated with miR-15a-3p. Overexpression experiments showed that and LUADT1 and miR-15a-3p did not significantly affect the expression of each other. Moreover, LUADT1 overexpression mediated the upregualtion of Twist1, and miR-15a-3p overexpression played an oppsoite role. Transwell assays showed that LUADT1 and Twist1 overexpression mediated the increased rate of cell invasion and migration, while miR-15a-3p overexpression mediated the decreased rate of cell invasion and migration. In addition, miR-15a-3p overexpression played an oppsoite role and attenuated the effects of LUADT1 overexpression. Therefore, LUADT1 may sponge miR-15a-3p to upregulate Twist1 in SCLC, thereby promoting cancer cell invasion and migration.

**Trial registration:**

2017GZH-1-201,746,382, registered at Jan 02,2017.

## Background

According to the lastest GLOBOCAN statistics, lung cancer is the most common malignancy and the leading cause of cancer deaths in both males and females [1, 2]. In 2018, lung cancer affected 2,093,876 new cases, which accounts for 11.6% of all cancer cases [1, 2]. It caused 1,761,007 new cases, which accounts for 18.4% of cancer-related mortalities [1, 2]. Small cell lung cancer (SCLC) possesses about 15% of all lung cancers [3]. More than 40 trials have been performed in 1970, but treatment outcomes of SCLC have not been significantly improved for decades [4]. As a conseuqence, more than 93% of SCLC patients will eventually die of this disease [4]. At present, molecular pathways involved in SCLC remain to be elusive and the development of targeted therapies is limited [5].

Accumulative evidence has shown that non-coding RNAs (ncRNAs), such as long non-coding RNAs (> 200 nt, lncRNAs) or microRNAs (~ 20 nt, miRNAs) are critical players in the development and progression of cancer [6–8]. ncRNAs encode no proteins but participate in cancer biology by regulating downstream gene expression [9]. Besides that, recent studies have also shown that lncRNAs can interact with miRNAs to regulate diverse pathological processes [10]. In a recent study, Qiu et al. reported a novel oncogenic lncRNA named LUADT1 in lung adenocarcinoma [11]. Our bioinformatics analysis showed that LUADT1 may form strong base pairing with miR-15a-3p, which can target Twist1 to suppress gastric cancer [12]. This study was therefore performed to analyze the interactions between LUADT1 and miR-15a-3p in SCLC.

## Methods

### SCLC patients and specimens

This study passed the review board of the First Affiliated Hospital of Guizhou University Ethics Committee. Research subjects of this study included 60 SCLC patients (gender: 34 males and 26 females; 37 to 65 years, 52.1 ± 6.3 years) who were selected from the 98 SCLC patients admitted to the aforementioned hospital between May 2017 and May 2019. The inclusion criteria were: 1) diagnosed by histopathological exams; 2) newly diagnosed cases. The exclusion criteria were: 1) recurrent SCLC; 2) therapies were initiated; 3) multiple clinical disorders were diagnosed. After admission, all SCLC patients were informed of the experimental principle. Informed consent was signed by all patients.

Lung biopsy was performed under the guidance of MRI before the initiation of therapies. During a biopsy, tumor (SCLC) and non-tumor tissue were collected form all patients. All tissue samples were tested by performing histopathological exams. According to the clinical findings, the 60 patients were staged based on AJCC standards. There were 27 and 33 cases at clinical stage III and IV, respectively.

### SCLC cell line and cell transfection

SHP-77 and H69 human SCLC cell lines (ATCC, USA) were used as the SCLC cell model. Cells were cultivated under conditions of 37 °C, 5% CO_2,_ and 95% humidity. Cell culture medium was a mixture of 10% FBS and 90% RPMI-1640 Medium. Cells were harvested at 80% confluence to perform cell transfections.

Negative control (NC) miRNA and miR-15a-3p mimic were from GenePharma (Shanghai, China). Vectors expressing LUADT1 and Twist1 were constructed using the pcDNA3.1 vector (GenePharma). Lipofectamine 2000 (GenePharma) was used to transfect 10 nM vectors (empty vector as NC group) or 40 nM miRNAs (NC miRNA as NC group) into 10^6^ SHP-77 cells. Cells were harvested at 24 h post-transfection to perform the following experiments. Untransfected cell was used as control (C) cells in all cases of transfections.

### RNA extraction and quantitative reverse transcription PCR (RT-qPCR)

To measure gene expression levels, SHP-77 cells (10^5^ cells harvested at 24 h post-transfection) and tissue samples (0.03 g tissue ground in liquid nitrogen) were subjected to total RNA extractions using Ribozol (Sigma-Aldrich). To harvest miRNAs, 85% of ethanol was used to precipitate RNA samples. All RNA samples were treated with DNase I for 2 h at 37 °C to digest genomic DNAs.

To measure the levels of LUADT1 and Twist1 mRNA expression, the MMLV Reverse Transcriptase kit (Lucigen) was used to perform all reverse transcriptions and SYBR Green Master Mix (Bio-Rad) was used to prepare qPCR assays with GAPDH as an endogenous control.

To measure the levels of mature miR-15a-3p expression, poly (A) addition, reverse transcriptions and all qPCR assays were performed using All-in-One™ miRNA qRT-PCR Detection Kit (Genecopoeia). The endogenous control was U6.

Three replicate reactions were set for each experiment and Ct values were processed using the 2^-ΔΔCT^ method.

### Western blot

Western blot was performed to explore the effects of transfections on the expression of Twist1 protein. At 24 h post-transfection, SHP-77 cells were harvested and total proteins were extracted from 10^5^ cells using RIPA solution (GenePharma) and protein concentrations were measured using bicinchoninic acid assay (BCA) assay (GenePharma). All samples were incubated with boiling water for 12 min to denature proteins, following by electrophoresis (10% SDS-PAGE gel) to separate proteins. PVDF membranes were used to perform protein transfer and blocking was achieved by incubating the membranes with 5% non-fat milk for 2 h at room temperature. Following that, membranes were first incubated with anti-Twist1 (1: 2000, ab50887, Abcam) anti-GLUT1 (1: 2000, ab15309, Abcam) rabbit primary antibodies for 15 h at 4 °C, followed by incubation with goat HRP (IgG) (1:2000; ab6721; Abcam) secondary antibody for 2 h at 25 °C. ECL Western Blotting Substrate Kit (ab65623, Abcam) was used to develop signals and grey values were processed using Image J v1.46 software.

### Transwell assays

Transwell assays were performed to analyze the effects of transfections on SHP-77 and H69 cell invasion and migration. Cells were counted after trypan blue staining. Serum-free RPMI-1640 Medium (1 ml) was mixed with 3 × 10^4^ cells to prepare single-cell suspensions. To perform Transwell assays, cell suspensions were injected into the upper chamber. In contrast, a mixture of 80% RPMI-1640 Medium and 20% FBS was added to the lower chamber. It is worth noting that membranes were coated by Matrigel (300μg/ml, Millipore, USA) for 6 h before invasion assay. The purpose is to mimic in vivo cell invasion. Transwell chambers were incubated under the aforementioned conditions for 16 h. After 0.1% crystal violet (Sigma-Aldrich, USA) staining for 12 min at room temperature, cells were observed under a light microscope and counted using Image J v1.46 software.

### Statistical analysis

All experiments were repeated 3 times and mean values were calculated. All statistical analyses were performed using mean values. Correlations were analyzed by Pearson’s Correlation Coefficient. Differences were explored by ANOVA (one-way) combined with the Tukey test (among different cell groups) or paired t-test (between two types of tissues). *p* < 0.05 was statistically significant.

## Results

### LUADT1 was upregulated in SCLC and may bind miR-15a-3p

**Fig. 1:**
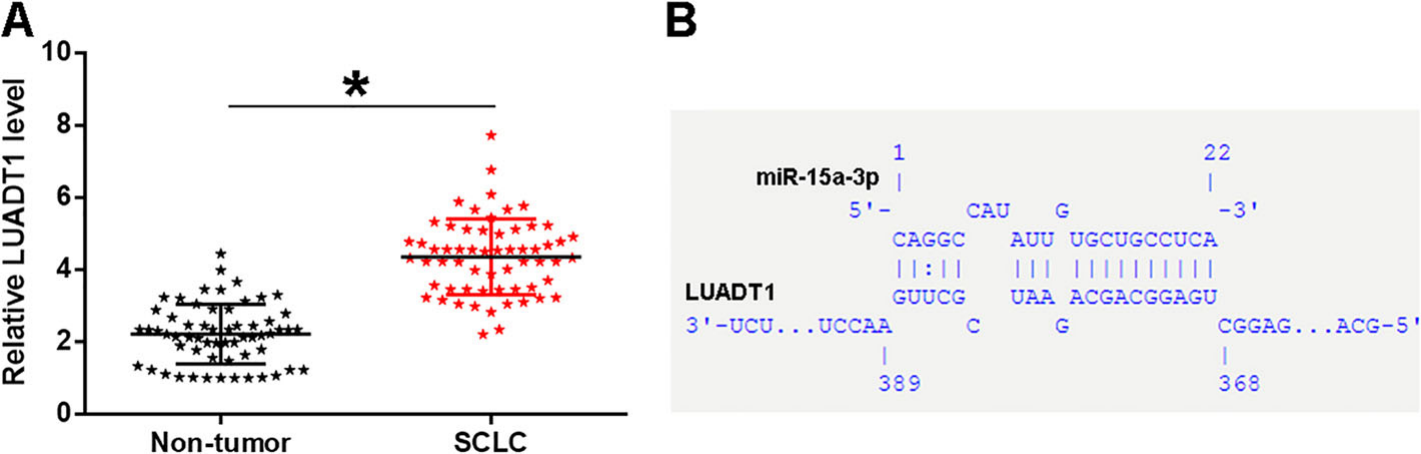
LUADT1 was upregulated in SCLC and might bind miR-15a-3p. Levels of LUADT1 in two types of tissues (SCLC and non-tumor) were measured and compared by qPCR and paired t-test, respectively (**a**). The interactions between LUADT1 and all unknown human miRNAs (available at http://www.mirbase.org/) were analyzed by IntaRNA (http://rna.informatik.uni-freiburg.de/IntaRNA/Input.jsp). It can be observed that LUADT1 may form the strongest interaction with miR-15a-3p among all human miRNAs (**b**). Data were expressed as the mean values of 3 replicates, *p < 0.05

Levels of LUADT1 in two types of tissues (SCLC and non-tumor) were measured and compared by qPCR and paired t-test, respectively. Comparing to non-tumor samples, significantly higher expression levels of LUADT1 were observed in SCLC tissues (Fig. 1a, *p* < 0.05). The interactions between LUADT1 and all unknown human miRNAs (available at http://www.mirbase.org/) were analyzed by IntaRNA (http://rna.informatik.uni-freiburg.de/IntaRNA/Input.jsp). It can be observed that LUADT1 may form the strongest interaction with miR-15a-3p among all human miRNAs (Fig. 1b).

### LUADT1 in SCLC was not correlated with miR-15a-3p but was positively correlated with its downstream target Twist1

**Fig. 2:**
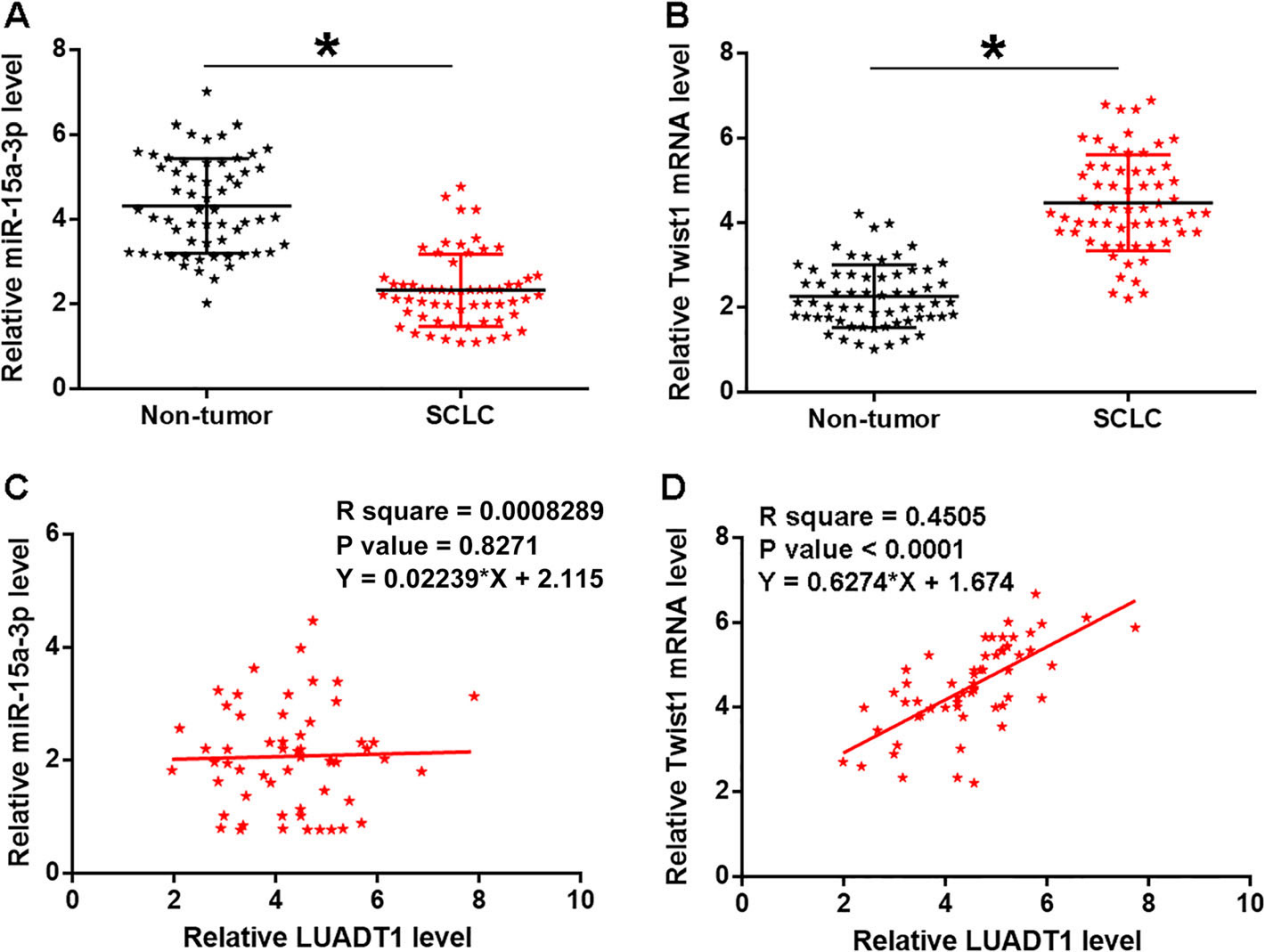
LUADT1 in SCLC was not correlated with miR-15a-3p, and positively correlated with its downstream target Twist1. Levels of miR-15a-3p (**a**) and Twist1 mRNA (**b**) in two types of tissues (SCLC and non-tumor) were measured and compared by qPCR and paired t-test, respectively. The correlation between LUADT1 and miR-15a-3p (**c**)/ Twist1 mRNA (**d**) across SCLC tissues was analyzed by Pearson’s Correlation Coefficient. Data were expressed as the mean values of 3 replicates, *p < 0.05

Levels of miR-15a-3p and Twist1 mRNA in two types of tissues (SCLC and non-tumor) were measured and compared by qPCR and paired t test, respectively. Comparing to non-tumor samples, significantly lower expression levels of miR-15a-3p (Fig. 2a), and significnatly lower expression levels of Twist1 mRNA (Fig. 2b) were observed in SCLC tissues (*p* < 0.05). The correlation between LUADT1 and miR-15a-3p/Twist1 mRNA was analyzed by Pearson’s Correlation Coefficient. It can be observed that LUADT1 was not significantly correlated with miR-15a-3p (Fig. 2c), but was positively correlated with Twist1 mRNA (Fig. 2d) across SCLC tissues.

### LUADT1 and miR-15a-3p failed to regulate the expression of each other

**Fig. 3:**
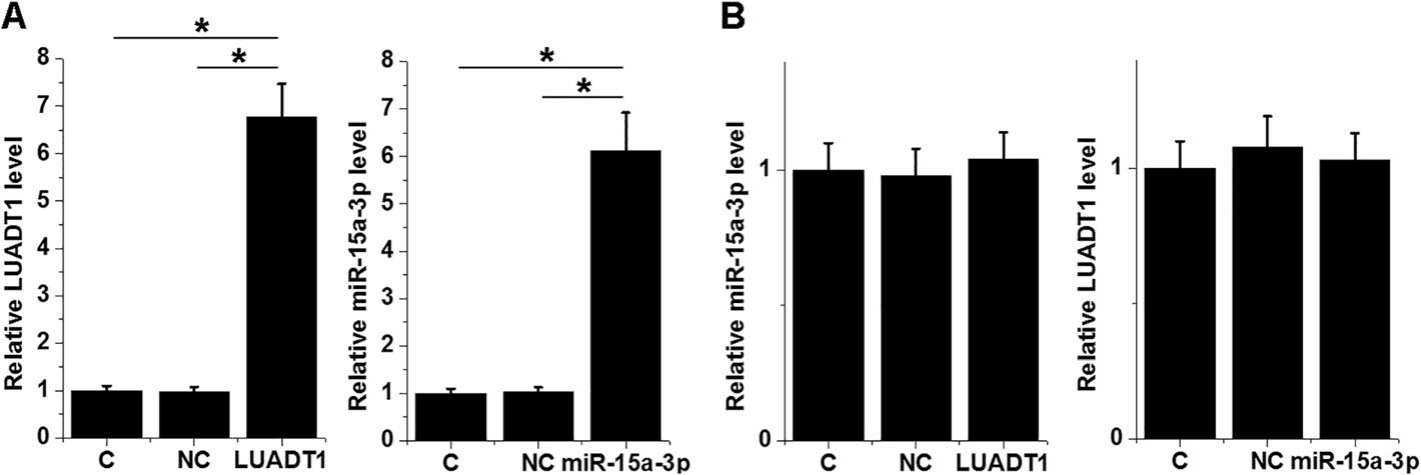
the expression of LUADT1 and miR-15a-3p is not correlated. SHP-77 cells were transfected with the LUADT1 expression vector and miR-15a-3p mimic. Overexpression of LUADT1 and miR-15a-3p was confirmed by qPCR at 24 h post-transfection (**a**). The interaction between LUADT1 and miR-15a-3p was also analyzed by qPCR (**b**). Data were expressed as the mean values of 3 replicates, *p < 0.05

SHP-77 cells were transfected with the LUADT1 expression vector and miR-15a-3p mimic. Overexpression of LUADT1 and miR-15a-3p was confirmed by qPCR at 24 h post-transfections. Comparing to C and NC (NC miRNA or empty pcDNA3.1) groups, expression levels of LUADT1 and miR-15a-3p were significantly upregulated (Fig. 3a, *p* < 0.05). Comparing to two controls, overexpression of LUADT1 and miR-15a-3p did not significantly affect the expression of each other (Fig. 3b).

### LUADT1 upregulated Twist1 through miR-15a-3p

**Fig. 4:**
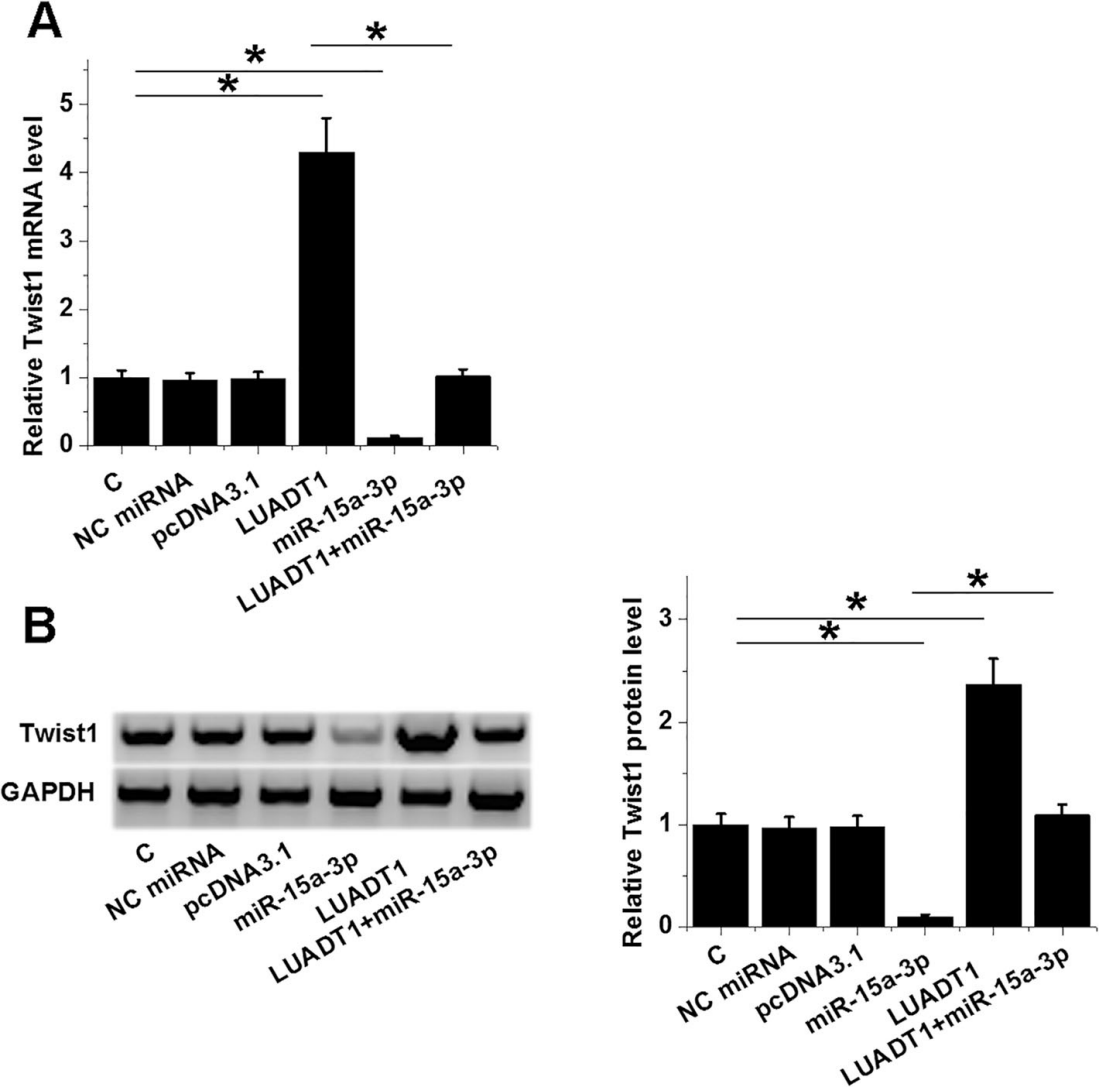
LUADT1 upregulated Twist1 through miR-15a-3p. The effects of LUADT1 and miR-15a-3p overexpression on the expression of Twist1 were analyzed by qPCR and western blot on both mRNA (**a**) and protein levels (**b**), respectively. Data were expressed as the mean values of 3 replicates, *p < 0.05

The effects of LUADT1 and miR-15a-3p overexpression on the expression of Twist1 were analyzed by qPCR and western blot at both mRNA (Fig. 4a) and protein levels (Fig. 4b), respectively. LUADT1 overexpression mediated the upregulation of Twist1. MiR-15a-3p overexpression played the opposite role and attenuated the effects of LUADT1 overexpression (*p* < 0.05).

### LUADT1 promoted cancer cell invasion and migration through Twist1 and miR-15a-3p

**Fig. 5:**
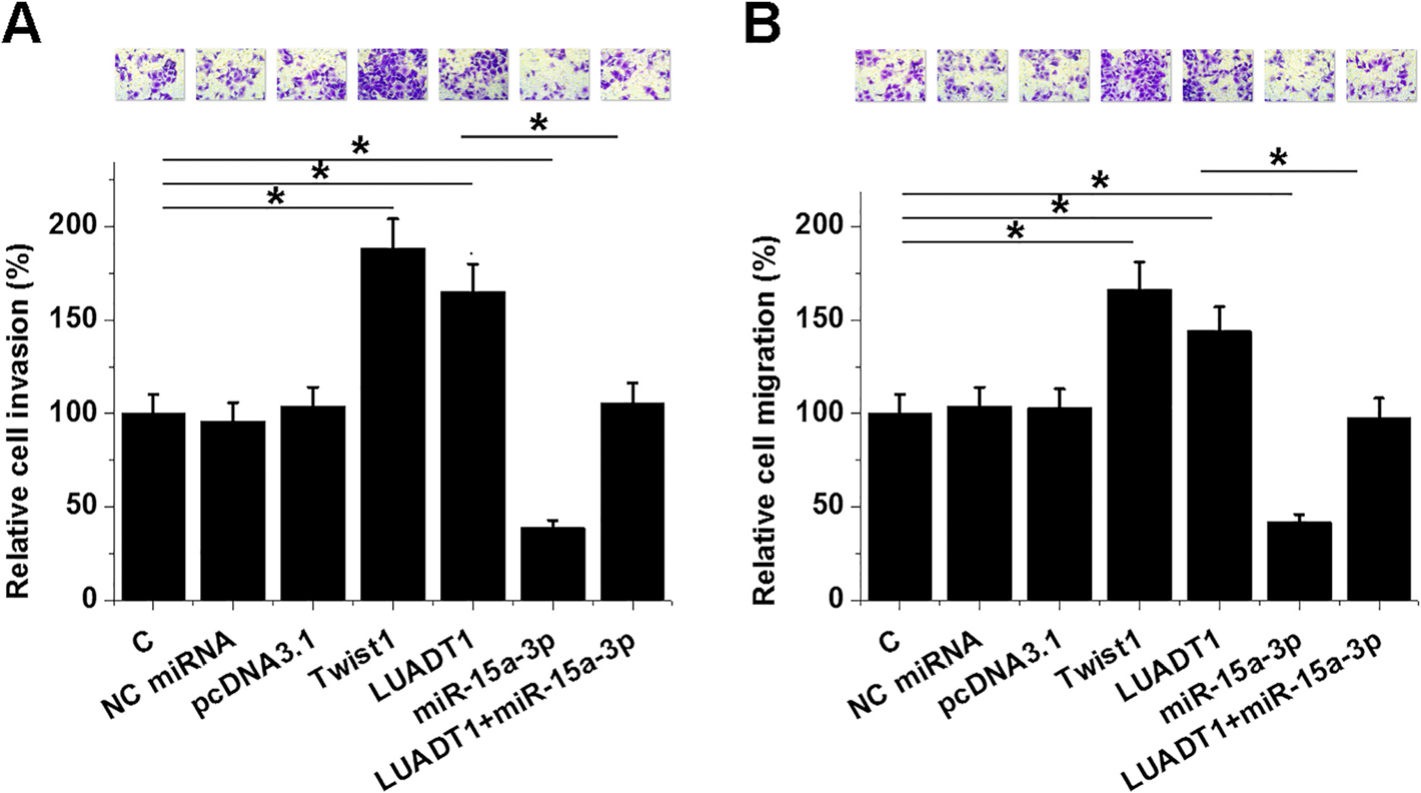
LUADT1 promoted cancer SHP-77 cell invasion and migration through Twist1 and miR-15a-3p. Transwell assays were performed to analyze the effects of transfections on the invasion (**a**) and migration (**b**) of SHP-77 cells. Data were expressed as the mean values of 3 replicates, *p < 0.05

Transwell assays were performed to analyze the effects of transfections on the invasion (Fig. 5a) and migration (Fig. 5b) of SHP-77 cells. Transwell assays showed that LUADT1 and Twist1 overexpression mediated the increased, while miR-15a-3p mediated the decreased rate of cell invasion and migration. In addition, miR-15a-3p overexpression played the opposite role and attenuated the effects of LUADT1 overexpression (*p* < 0.05). To further confirm the roles of LUADT1, miR-15a-3p, and Twist1 in regulating SCLC cell invasion and migration, Transwell assays were repeated using H6the 9 SCLC cell line. Similarly, LUADT1 and Twist1 overexpression mediated the promoted, while miR-15a-3p mediated the inhibited cell invasion (Additional file 1: Figure S1A) and migration (Additional file 1: Figure S1B). In addition, miR-15a-3p overexpression played the opposite role and inhibited the effects of LUADT1 overexpression (*p* < 0.05).

## Discussion

The function of LUADT1 in SCLC was investigated in this study. We found that LUADT1 was upregulated in NSCLC and regulated cancer cell invasion and migration. We also provided evidence that LUADT1 may sponge miR-15a-3p to upregulate Twist1, thereby promoting cancer cell invasion and migration.

LUADT1 plays an oncogenic role in lung adenocarcinoma [11]. In lung adenocarcinoma, LUADT1 regulates the expression of p27 through epigenetic pathways to promote cancer cell proliferation [12]. Lung adenocarcinoma is a type of non-small cell lung cancer (NSCLC), which has a different pathogenesis to that of SCLC [13]. Although it is well known that NSCLC and SCLC require the involvement of different genetic factors [13], the transformation from NSCLC to SCLC is frequently observed [14]. In this study, we found that LUADT1 was also upregulated in SCLC and could promote the invasion and migration of cancer cells. Therefore, LUADT1 may have oncogenic functions in both NSCLC and SCLC.

MiR-15a-3p has been characterized as a tumor-suppressive miRNA in several types of cancers, such as ovarian cancer [15] and prostate cancer [16]. Overexpression of miR-15a-3p resulted in inhibited cell proliferation, invasion, and migration [15, 16]. In a recent study, Wang et al. reported that miR-15a-3p could negatively regulate Twist1 to suppress gastric cancer [12]. Twist1 can induce cancer metastasis through multiple ways, such as epithelial-mesenchymal transition and the upregulation of discoidin domain receptor 2 [17, 18]. In this study, we also observed the downregulation of Twist1 after miR-15a-3p overexpression. Therefore, Twist1 is also regulated by miR-15a-3p in SCLC.

Interestingly, our bioinformatics analysis revealed a strong interaction between miR-15a-3p and LUADT1, while overexpression experiments revealed no significant expression regulation by each other. Therefore, LUADT1 is unlikely a target of miR-15a-3p. Instead, LUADT1 is likely a molecular sponge of miR-15a-3p. This speculation is supported by the observation of the upregulation of Twist1 after LUADT1 overexpression.

## Conclusion

In conclusion, LUADT1 is upregulated in SCLC. In addition, LUADT1 may sponge miR-15a-3p to upregulate Twist1, thereby promoting cancer cell invasion and migration.

## Supplementary information

**Additional file 1: Figure S1.** LUADT1 promoted H69 cell invasion and migration through Twist1 and miR-15a-3p.Transwell assays were also carried out to explore the effects of transfections on the invasion (A) and migration (B) of H69 cells. Data were expressed as the mean values of 3 replicates, *p < 0.05.

## Data Availability

The analyzed data sets generated during the study are available from the corresponding author on reasonable request.

## Abbreviations

BCA: Bicinchoninic acid assay
C: Control
FBS: Fetal bovine serum
LncRNA: Long non-coding RNA
LUADT1: Lung adenocarcinoma associated transcript 1
NC: Negative control
RT-qPCR: Quantitative reverse transcription PCR
SCLC: Small cell lung cancer
