# Supplementary figures and images for: LncRNA LUADT1 sponges miR-15a-3p to upregulate Twist1 in small cell lung cancer
Source: BMC Pulm Med. 2019 Dec 16;19:246. doi: 10.1186/s12890-019-0991-7 (PMC6915879; doi:10.1186/s12890-019-0991-7)

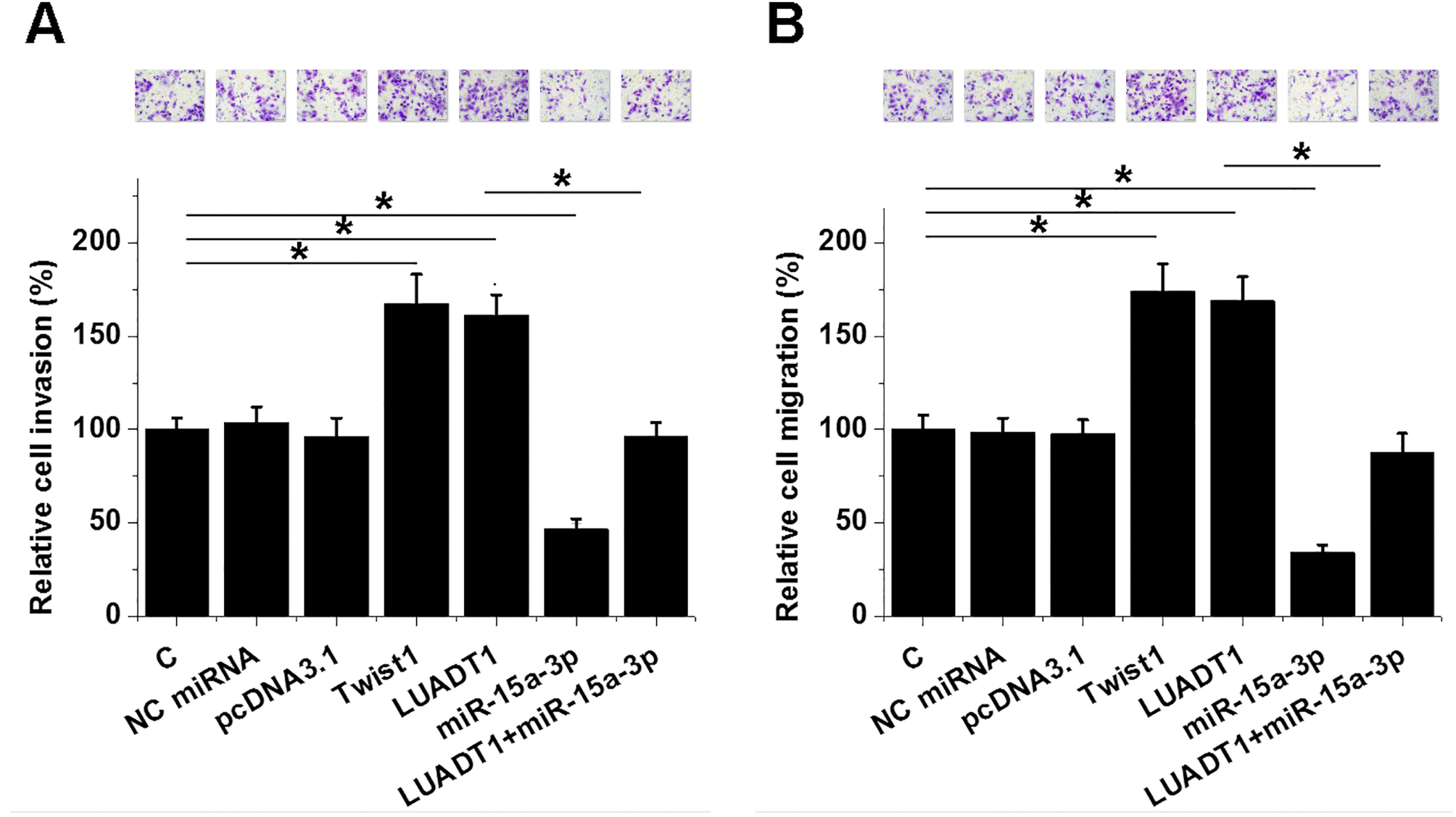

Supplement: Supplementary file 1 — Additional file 1: Figure S1. LUADT1 promoted H69 cell invasion and migration through Twist1 and miR-15a-3p.Transwell assays were also carried out to explore the effects of transfections on the invasion (A) and migration (B) of H69 cells. Data were expressed as the mean values of 3 replicates, *p < 0.05. [file 12890_2019_991_MOESM1_ESM.jpg]
